# Potentially inappropriate medications for the elderly: Incidence and impact on mortality in a cohort ten-year follow-up

**DOI:** 10.1371/journal.pone.0240104

**Published:** 2020-10-28

**Authors:** Natacha Christina de Araújo, Erika Aparecida Silveira, Brenda Godoi Mota, João Paulo Neves Mota, Ana Elisa Bauer de Camargo Silva, Rafael Alves Guimarães, Valéria Pagotto

**Affiliations:** 1 Faculty of Nursing, Postgraduate Program in Nursing, Federal University of Goiás, Goiânia, Goiás, Brazil; 2 Faculty of Medicine, Postgraduate Program in Health Sciences, Federal University of Goiás, Goiânia, Goiás, Brazil; 3 Faculty of Nursing, Federal University of Goiás, Goiânia, Goiás, Brazil; Hong Kong Polytechnic University, HONG KONG

## Abstract

**Introduction:**

Pharmacological therapy plays an important role in disease control in the elderly; unfortunately, this comes with a high prevalence in the use of medications classified as potentially inappropriate.

**Objective:**

To analyze the incidence, risk factors, and survival of elderly people using potentially inappropriate medications (PIM).

**Method:**

A ten-year follow-up assessment of elderly participants residing in a capital of Central Brazil was conducted. The initial assessment (baseline) included 418 elderly people. Data were collected through home interviews guided by a questionnaire covering socioeconomic, demographic, living conditions, and health variables. The medication information obtained comprised active ingredient, dosage, route, and regimen for the medications. The PIMs were classified according to 2019 Beers Criteria. The analyses were performed using STATA 15.0. For survival analysis, a Cox Regression was performed with the respective Kaplan Meier curve.

**Results:**

The incidence of PIM was 44.1 cases (95% CI: 35.2–54.7) per 1,000 people a year. The most used PIMs were nifedipine, glibenclamide, and sodium diclofenac. The risk factors were polypharmacy (aRR: 3.00; 95% CI: 1.31–6.88) and diabetes mellitus (aRR: 1.57; 95% CI: 1.03–2.39). We identified no statistically significant association between survival and the use of PIM.

**Conclusion:**

The study highlights the high consumption of PIM among the elderly causing polypharmacy risks. Health professionals working in drug treatment need to be alert to polypharmacy risks to ensure the rational use of medications to prevent adverse reactions and other health problems.

## Introduction

Medications are a therapeutic resource used by all age groups within the population [1] that has contributed to increased life expectancy and an aging population. Despite the benefits of using medications by the elderly, the simultaneity of chronic diseases leads to consumption of multiple medications (polypharmacy) [2] and self-medication [3, 4]. In view of these practices, it is important to monitor the effectiveness and safety of the medications used, given that some are classified as potentially inappropriate medications (PIM) for the elderly [5, 6].

Studies on the use of PIM have been conducted in Brazil [2, 7–9] and worldwide [10–15] indicating an estimated prevalence ranging from 21% to 84% [7–17]. The use of PIM increases the occurrence of adverse reactions and events, which may increase the risk of death. However, the relationship between the use of PIM and mortality in the elderly remains controversial [18–21].

Some of the factors associated with the use of PIM are chronic diseases [2], polypharmacy [2–4], being female [7], and an increased use of health services [9].

The Beers Criteria is used to classify PIM; the list is constantly updated, with new insights into medications that can interfere with and cause divergences to their expected outcomes. To date, two studies have relied on the Beers 2019 criteria [22, 23]. One study involved a three-month follow-up and identified that PIMs cause mortality in hospitalized elderly [22] and the other study investigated PIM use in a community-dwelling population [23]. Although some studies have analyzed the use of PIM longitudinally [24, 25], most are cross-sectional [7, 8, 16, 17, 26–31], which precludes establishing the causal relationship.

The trend of our aging population in conjunction with the already high consumption of PIM has resulted in constant updates of the PIM evaluation criteria available in the literature. The primary objective of our study is to analyze the incidence and assess the risk factors involved in PIM use in a community-dwelling elderly cohort. Our secondary objective is to investigate the survival of elderly people using PIM.

## Materials and methods

### Study design and participants

This study used data from the *ProjetoIdososGoiânia*, a prospective cohort study conducted in the city of Goiânia, Goiás, the capital of central-west Brazil. Sampling and data collection details have previously been published [32, 33]. This study began in 2008 (*baseline*), it included 418 elderly (people aged 60 or over) living in the community utilizing the Unified Health System. The project was approved by the Research and Ethics Committee of Clinics Hospital of Federal University of Goiás (Protocol n° 2.500.0441/2018) and all subject gave informed consent and they anonymity was preserved.

### Follow-up and data collection

The follow-up assessment of the 418 elderly people from the *baseline* study was performed after ten years. In 2018, deaths were identified using the Mortality Information System (MIS), and variables included: name of the elderly person, date of birth and address. Any divergences in the identification were crosschecked using other local information systems and/or by searching the original address, or by searching the neighborhood itself (neighborhood and Basic Health Unit), or using the additional data contained in the basic questionnaire (phones, contact with other relatives). These procedures were adopted to identify possible contradictions in the data that could prevent death certificate confirmation in the MIS. All deaths declared in the MIS were also confirmed using the elderly person's address and crosschecked with family members and/or neighbors.

Of the 418 elderly people in the original sample, 34.9% were deceased (n = 146). The survivors undertook a follow-up assessment. The assessment included elderly with cognitive capacity (this was assessed using the Mini-Mental State Examination [34]). We only included elderly people who we were able to contact (present at home within five attempted visits). Among them, 6% refused to answer the questionnaire (n = 25) and 6% (n = 25) were not contactable. A total of 221 elderly people undertook the follow-up assessment.

Baseline and follow-up data were used in this study. Data collection was performed, both in 2008 and in 2018, at the elderly person's home, through the application of a standardized questionnaire, containing information about medication use, demographic, economic conditions, health conditions, and lifestyle.

In situations where the elderly person presented cognitive impairment, a legal guardian answered the objective health questions. People were excluded from the follow-up assessment if the elderly person was not home after at least five attempts, the address was not found during the visit, or the elderly person had moved to another city.

### Study variables

#### Outcome variables

Our primary objective was to investigate the use of PIM. To investigate the use of PIM, we initially asked the individual about the medications they used daily, “Are you taking any medication?” When medication was confirmed, the elderly (or their caregiver) was asked to provide all the drugs they use with the respective prescriptions and/or packaging. We then collected further information including the name of the medication, dosage, form, and the number of medications used.

Based on the use of medications information, we classified PIM use according to the 2019 Beers Criteria [5]. The Beers Criteria is subdivided into four sections: 1. medications for elderly people out of palliative care; 2. medications for elderly with specific syndromes, to be used with caution, 3. medications with risks of drug interactions; 4. medications to avoid and/or adjust doses based on kidney function. For the analysis of this study, the first section is used, which refers to medications for elderly people outside of palliative care. This section was selected in isolation because the Beers Criteria is explicit; this section allows analysis of the medication in isolation, without the need to analyze clinical conditions.

The secondary outcome of this study was to investigate the survival status of any elderly confirmed using the MIS. For the participants who died prior to the ten-year follow-up, the period from the first investigation (*baseline*) to the date of death was recorded.

#### Independent variables

For the survival analysis, death was considered an outcome. The independent variables were subdivided further. The first section involved sociodemographic details such as: gender, age (stratified into age groups of 70–79 and ≥80 years), education (illiterate, 1–4, 5–8, and ≥9 years of study), race/color (white, mixed, and black), marital status (with or without partner), and economic class (A/B/C and D/E). The second section investigated health conditions (self-rated health), number of diseases, polypharmacy, arterial hypertension, diabetes mellitus, and nutritional status.

Economic class was classified using the Brazil Economic Classification Criterion (CCEB) consisting of data such as level of education and items owned by the family. Economic stratification corresponds with the economic class of the elderly person (A, B, C, D, and E). For statistical purposes, the classification was redefined as A/B/C, and D/E [35], with classes D/E corresponding to elderly of low economic status.

Self-rated health was assessed with the question: “How do you consider your health status?” The responses were categorized into very good/good/fair and poor/very poor [36].

The number of diseases was assessed based on the response to the question: “What diseases did the doctor say you have?” Responses were classified according to the quantity reported by the participant.

Arterial hypertension was assessed using a self-report, as well as blood pressure measurement and medication use. Blood pressure was taken using an OMROM semiautomatic device on the elderly's left arm while in a sitting position with three replicates (statistical analyses are completed using the mean). The reference values were the systolic and diastolic pressure values above 140 mmHg and 90 mmHg, respectively [37].

Diabetes mellitus was assessed by self-report and/or the use of medications and/or glycemic control. Fasting blood glucose results ≥ 126 mg/dl and/or glycated hemoglobin ≥ 7% were considered elevated [38].

Hospitalization in the previous year was identified through the question: “Did you stay in the hospital last year?” Polypharmacy was defined as taking five or more medications [10, 39].

Nutritional status was assessed using the body mass index (BMI) kg/m^2^ estimated by the formula: weight (kg)/height^2^ (m^2^). Weight was measured using a Tanitabrand electric scale (200 kg capacity and 100 g precision). Height was measured using a wall stadiometer (precision of 0.1 cm). Two replicates were collected and the means were used for our analyses. For the classification of BMI, the Lipschitz criteria (1994) [40] were used, which considers the typical changes in body composition with aging with each participant classified as: low weight (BMI < 22 kg/m^2^), eutrophic (BMI between 22 and 27 kg/m^2^), and overweight (BMI > 27 kg/m^2^).

### Statistical analysis

Data were analyzed using STATA version 15.0. The incidence rate (IR) of PIM use was estimated using the ratio between the number of new cases of PIM use and the total number of persons-time in the at-risk population with follow-up multiplied by 1,000. The at-risk population consisted of the total number of elderly people interviewed in the cohort after 10 years, excluding those participants who already used PIM in the baseline or died before the follow-up assessment (between 2008 and 2018).

To analyze the factors associated with PIM use, a generalized linear model was constructed, using a bivariate analysis between the dependent variable (use of PIM) and each independent variable; this provided us the crude relative risk (cRR) with a 95% confidence interval (95% CI). Next, variables with a *p*-value < 0.20 were included in a multiple regression model with a robust variance. The results of the regression model are presented as the adjusted relative risk (aRR), with 95% CI. The quality of the regression model was estimated using a Pearson’s goodness-of-fit test. Variables with *p*-values < 0.05 in the final model were considered statistically significant.

The mortality rate was calculated using the ratio between the number of deaths and total person-time in the cohort multiplied by 1,000. Next, a bivariate and multivariate analysis was performed to test the study hypothesis. We hypothesized that elderly people using PIM present a lower survival [S] (or higher mortality rate) than elderly people not using PIM in the baseline assessment, or, the S of elderly using PIM is less than the S of elderly not using PIM. Initially, a Cox bivariate analysis was performed between the dependent variable (survival in the elderly) and the independent variable (using PIM). This analysis provided us the crude hazard ratio (cHR) and respective 95% CI as the measure of effect. Next, a Cox proportional regression model was adjusted to verify the potential association between the study hypothesis variable (use of PIM) and survival. Potential confounding variables were adjusted in the model: sex, age group, socioeconomic class, and polypharmacy. The results of the regression model are presented as adjusted hazard ratio (aHR), with 95% CI. Finally, survival curves were plotted for elderly people with and without the use of PIM via the Kaplan-Meier (KM) estimator. Results with *p*-values < 0.05 in the final model were considered statistically significant.

## Results

### Baseline characteristics

Analysis of the 418 elderly people in the baseline assessment identified that 66.0% of individuals in the study were women, the mean age was 70.6 years (with a standard deviation of 7.1 years), 54.8% were married, and 71.1% had less than four years of formal study. The health conditions of participants identified 27.2% believing their health was poor/very poor, 24.4% hospitalized in the previous year, 27.5% used polypharmacy, and 43.5% presented more than three diseases. Of the diseases, the most prevalent were arterial hypertension (60.3%) and diabetes mellitus (23.4%) (Table 1).

**Table 1:** Descriptions of the elderly participants according to sociodemographic and health variables recorded in the baseline assessment, Central-Brazil, 2008.

Variables	n*	%
**Sex**		
Female	276	66.0
Male	142	34.0
**Age group (years)**		
60–69	203	48.6
70–79	168	40.2
≥80	47	11.2
**Race/skin color (self-report)**		
White	194	46.4
Mixed	178	42.6
Black	46	11.0
Years of study**		
0–4	266	71.1
> 4	108	28.9
**Marital status**		
Lives with partner	229	54.8
Lives without partner	189	45.2
**Economic class**		
A/B/C	259	62.0
D/E	159	38.0
Self-perception of health**		
Very good / good / regular	300	72.8
Poor / very poor	112	27.2
**Hospitalization in previous year**	102	24.4
**Number of diseases** ≥ 3	182	43.5
**Arterial hypertension (yes)**	252	60.3
**Diabetes mellitus (yes)**	98	23.4
**Polypharmacy**	115	27.5
**Nutritional status**		
Low weight	66	15.8
Eutrophic	147	35.2
Overweight	205	49.0

*n = 418

**Missing data = 44

***Missing data = 6.

In the baseline assessment, 365 (87.2%) participants consumed medication. The mean number of medications used was 3.3 (standard deviation of 2.6), varying from 1 to 12 (data not shown). Of the 365 participants who used medication, 183 (50.1%) used PIM, the most frequently being nifedipine (24.0%), glibenclamide (20.2%), and sodium diclofenac (13.7%), as shown in Table 2.

**Table 2:** Distribution data of the elderly participants according to types of PIM used in the baseline assessment, according to the Beers Criteria, Central-Brazil, 2008.

Medications	n*	%
**Central Alpha-Agonists**		
Nifedipine	44	24.0
Amiodarone	21	11.5
Digoxin	12	6.5
Methyldopa	12	6.5
Clonidine	6	3.3.
**Long-acting sulfonylureas**		
Glibenclamide	37	20.2
Glimepiride	8	4.4
**Nonselective NSAIDs for cyclooxygenase**		
Diclofenac	25	13.7
Meloxicam	5	2.7
Naproxen	2	1.1
Piroxicam	2	1.1
Ibuprofen	1	0.5
Ketoprofen	1	0.5
Etodolac	1	0.5
**Benzodiazepines (short, intermediate, and long acting)**		
Clonazepam	14	7.5
Diazepam	5	2.7
Alprazolam	2	1.1
Chlordiazepoxide	1	0.5
Lorazepam	1	0.5
**Musculoskeletal relaxants**		
Carisoprodol	14	7.6
Cyclobenzaprine	7	3.8
Orphenadrine	1	0.5
**Antidepressants**		
Amitriptyline	15	8.2
Paroxetine	3	1.6
Clomipramine	2	1.1
Nortriptyline	1	0.5
**Androgens**		
Estradiol	4	2.2
Estrogen	3	1.6
Regular insulin	1	0.5
**Peripheral Alpha 1-blockers to treat hypertension**		
Doxazosin	5	1.4
**Antihistamines, 1**^**st**^ **Generation**		
Dexchlorpheniramine	2	1.1
Dimenhydrinate	1	0.5
Hydroxyzine	1	0.5
**Anti-spasmodics**		
Atropine	2	1.1
Scopolamine	1	0.5
**Antipsychotics, 1**^**st**^**and 2**^**nd**^ **generation**		
Phenobarbital	3	1.6
**Antiparkinsonian Agents**		
Trihexyphenidyl	1	0.5
**Anti-infectious**		
Nitrofurantoin	1	0.5
**Nitro-thrombotic**		
Dipyridamole	1	0.5
**Gastrointestinal**		
Metoclopramide	1	0.5
**Total elderly using PIM**	**183**	100.0
**Total types of PIM**	**40**	

*n = 118.

### Incidence and risk factors associated with PIM

The cohort population consisted of 127 elderly people who completed the follow-up assessment after ten years. We excluded participants who died during the period, those not able to be contacted, and those who already used PIM in the baseline assessment. Consequently, this resulted in 1,270 person-years.

There were 56 new cases of PIM use in the cohort. Thus, the incidence of PIM use in the sample was 44.1 cases (95% CI: 35.2–54.7) per 1,000 person-years (56 incident cases/1,270 person-years). Among the elderly who used PIM on in the follow-up assessment, the most popular consumed were diclofenac (25.0%), carisoprodol (19.6%), and orphenadrine (14.3%) (Table 3).

**Table 3:** Distribution of the elderly according to types of PIM used in the follow-up assessment, according to the Beers Criteria, Central-Brazil, 2018.

Medications	n*	%
**Central Alpha-Agonists**		
Nifedipine	5	8.9
Amiodarone	4	7.1
**Long-acting sulfonylureas**		
Glibenclamide	2	3.6
Glimepiride	2	3.6
**Nonselective NSAIDs for cyclooxygenase**		
Diclofenac	14	25.0
Meloxicam	1	1.8
Piroxicam	1	1.8
Ibuprofen	1	1.8
Ketoprofen	1	1.8
Etodolac	1	1.8
**Benzodiazepines (short, intermediate, and long acting)**		
Clonazepam	1	1.8
Alprazolam	3	5.3
Lorazepam	1	1.8
**Musculoskeletal relaxants**		
Carisoprodol	11	19.6
Cyclobenzaprine	6	10.7
Orphenadrine	8	14.3
**Antidepressants**		
Amitriptyline	1	1.8
Nortriptyline	1	1.8
**Androgens**		
Insulin	1	1.8
**Peripheral Alpha 1-blockers to treat hypertension**		
Doxazosin	6	10.7
**Antihistamines, 1st Generation**		
Dexchlorpheniramine	7	12.5
Dimenhydrinate	2	3.6
Hydroxyzine	4	7.1
Cyproheptadine	1	1.8
Clemastine	1	1.8
Chlorpheniramine	2	3.6
Promethazine	4	7.1
**Anti-spasmodics**		
Scopolamine	6	10.7
**Total elderly using PIM**	**56**	100.0
**Total types of PIM**	**28**	

Table 4 provides the results of the follow-up and the risk factors in the bivariate analysis. The incidence rate of PIM use was statistically higher in the elderly with diabetes mellitus (cRR: 1.87; 95% CI: 1.23–2.83), in polypharmacy use (cRR: 3.58; CI 95%: 1.56–8.22), and with three or more comorbidities (cRR: 1.51; 95% CI: 1.04–2.22).

**Table 4:** Bivariate analysis of the potential factors associated with the incidence of PIM use in the elderly cohort, Central-Brazil, 2008–2018.

Variables	Total (n = 127)	Incidence		cRR (95% CI)	*p*-value**
		n = 56	IR* (95% CI)		
**Sex**					
Female	75	29	38.7 (28.2–52.0)	1.00	
Male	52	27	51.9 (37.4–70.6)	1.34(0.91–1.98)	0.137
**Age group (years)**					
70–79	76	37	48.7 (36.8–63.4)	1.00	
≥ 80	51	19	37.3 (25.1–53.6)	0.76(0.50–1.17)	0.218
**Race/skin color**					
White	54	25	46.3 (32.9–63.7)	1.00	
Mixed	60	23	38.3 (26.8–53.4)	0.83(0.54–1.28)	0.392
Black	13	8	61.5 (32.9–106.7)	1.33(0.78–2.23)	0.282
**Years of study**					
0–4	81	33	40.7 (29.8–54.5)	1.00	
> 4	44	22	50.0 (33.9–71.4)	1.23 (0.82–1.83)	0.312
**Economic class**					
A/B/C	101	46	45.5 (35.1–58.2)	1.00	
D/E	26	10	38.5 (20.9–65.2)	0.84 (0.50–1.44)	0.534
**Marital status**					
Lives with partner	76	34	44.7 (32.9–59.6)	1.00	
Lives without partner	51	22	43.1 (29.2–61.6)	0.96 (0.64–1.44)	0.860
**Hospitalization**					
No	101	41	40.6 (30.8–52.7)	1.00	
Yes	26	15	57.7 (34.9–88.8)	1.42 (0.95–2.13)	0.090
**Self-perception of health**					
Very good/good/regular	106	44	41.5 (31.8–53.4)	1.00	
Poor/very poor	26	12	46.2 (26.6–74.8)	1.38 (0.89–2.13)	0.150
**Number of diseases**					
1–2	89	34	38.2 (28.1–50.9)	1.00	
≥ 3	38	22	57.9 (39.2–82.7)	1.51 (1.04–2.22)	**0.032**
**Polypharmacy**					
No	33	5	15.2 (0.6–31.8)	1.00	
Yes	94	51	54.3 (42.4–68.5)	3.58 (1.56–8.22)	**0.003**
**Arterial hypertension**					
No	56	19	33.9 (22.2–49.8)	1.00	
Yes	71	37	52.1 (38.9–68.6)	1.54 (0.99–2.36)	0.050
**Diabetes mellitus**					
No	118	49	41.5 (32.3–52.7)	1.00	
Yes	9	7	77.8 (36.5–146.0)	1.87 (1.24–2.83)	**0.003**
**Hypercholesterolemia**					
No	107	47	43.9 (33.9–56.0)	1.00	
Yes	19	9	47.4 (24.7–82.6)	1.08 (0.64–1.82)	0.777
**Nutritional status**					
Low weight	40	15	37.5 (23.1–57.7)	1.00	
Eutrophic	22	12	54.6 (31.5–88.4)	1.45 (0.84–2.53)	0.186
Overweight	65	29	44.6 (31.9–60.8)	1.19 (0.73–1.93)	0.483

cRR: Crude Relative Risk; 95% CI: 95% Confidence Interval

*Incidence rate per 1,000 person-year

**Wald chi-square test.

The final regression model analyzing the risk factors for the use of PMI was adjusted for sex, age group, self-rated health, polypharmacy, diabetes mellitus, arterial hypertension, number of morbidities, hospitalization, and nutritional status. The model represented an excellent fit (Pearson goodness-of-fit: χ^2^: 75.57; *p*-value = 0.99). In addition, multicollinearity was verified through a correlation matrix analysis of the variables and FIV test. In the correlation matrix, no correlations suggested multicollinearity between variables (Pearson's correlation coefficient [r] = - 0.069 between sex and self-rated health and r = + 0.234 is the coefficient between morbidities and diabetes mellitus). In the correlation matrix, there were low correlation coefficients between the number of morbidities and polypharmacy (r = 0.191), polypharmacy and hypertension (r = 0.197), and polypharmacy and diabetes (r = 0.094). The FIV test did not show multicollinearity between the variables (FIV < 4.0), with FIV ranging from 1.07 for diabetes to 1.46 for nutritional status (data not shown).

Table 5 summarizes the final regression model analysis of PIM use risk factors. The PIM use risk factors in the cohort were polypharmacy (aRR: 3.00; 95% CI: 1.31–6.88) and diabetes mellitus (aRR: 1.57; 95% CI: 1.03–2.39). These results indicate that the participant’s risk associated with using PIM was three times higher in individuals who consumed more than five drugs (polypharmacy) and 1.57 times higher in those diagnosed with diabetes mellitus.

**Table 5:** Multiple regression model of the factors associated with the incidence of potentially inappropriate medications (PIM) in the elderly cohort, Central-Brazil, 2008–2018.

Variables	aRR*	95% CI	p-value**
**Sex**			
Female	1.00		
Male	1.25	0.85–1.82	0.258
**Age group (years)**			
70–79	1.00		
≥ 80	0.73	0.49–1.09	0.120
**Self-perception of health**			
Very good/good/regular	1.00		
Poor/very poor	1.09	0.70–1.67	0.705
**Polypharmacy**			
No	1.00		
Yes	3.00	1.31–6.88	**0.009**
**Diabetes mellitus**			
No	1.00		
Yes	1.57	1.03–2.39	**0.036**
**Arterial hypertension**			
No			
Yes	1.30	0.85–1.99	0.219
**Hospitalization**			
No	1.00		
Yes	1.24	0.79–1.95	0.350
**Number of diseases**			
1–2	1.00		
≥ 3	1.07	0.84–1.55	0.725
**Nutritional status**			
Low weight	1.00		
Eutrophic	1.42	0.83–2.45	0.202
Overweight	1.00	0.63–1.60	0.990

aRR: adjusted relative risk; 95% CI: 95% confidence interval

* Poisson regression model adjusted for sex, age group, self-rated health, polypharmacy, diabetes mellitus, arterial hypertension, number of diseases, hospitalization, and nutritional status

**Wald's chi-square test.

#### Mortality and the use of PIM

The overall mortality rate observed in the sample was 41.4 deaths (95% CI: 36.0–47.3) per 1,000 person-years. The mortality rate in the group that already used PIM in the baseline assessment was 46.3 deaths (95% CI: 37.8–56.1) (70/1,513 person-years). In the unexposed group (who did not already use PIM in the baseline assessment) there were 37.7 deaths (95% CI: 31.0–45.4) (75/1,992 person-years). The bivariate analysis revealed no statistical difference between groups (cHR: 1.23; 95% CI: 0.89–1.70; *p*-value = 0.210).

A Cox regression model for survival analysis verified the potential association between the use of PIM and elderly survival. The model was adjusted according to age group, sex, economic class, polypharmacy, arterial hypertension, diabetes mellitus, number of diseases, and use of PIM. The model demonstrated that mortality was statistically higher in men (aHR: 1.02; 95% CI: 1.01–2.00). Mortality was also statistically higher in participants aged 70 to 79 (aHR: 1.60; 95% CI: 1.09–2.33), and participants aged 80 years or older (aHR: 4.06; 95% CI: 2.57–6.42). Mortality was also statistically higher in participants in the economic class D/E (aHR: 1.67; 95% CI: 1.67–2.35), those with diabetes mellitus (aHR: 2.22; 95% CI: 1.30–3.78), and those using polypharmacy (aHR: 1.98; 95% CI: 1.30–3.01) (see Table 6).

**Table 6:** Multiple regression model of factors associated with survival in the elderly cohort, Central-Brazil, 2008–2018.

Variables	aHR*	95% CI	p-value**
**Sex**			
Female	1,00		
Male	1.42	1.01–2.00	0.044
**Age group (years)**			
60–69	1,00		
70–79	1.60	1.09–2.33	**0.015**
≥ 80	4.06	2.57–6.42	**<0.001**
**Economic class**			
A/B/C	1.00		
D/E	1.67	1.19–2.35	**0.003**
**Polypharmacy**			
No	1,00		
Yes	1.98	1.30–3.01	**0.001**
**PMI use**			
No	1,00		
Yes	1.12	0.72–1.72	0.607
**Arterial hypertension**			
No	1.00		
Yes	0.87	0.61–1.23	0.435
**Diabetes mellitus**			
No	1.00		
Yes	2.22	1.30–3.78	**0.003**
**Number of diseases**			
1–2	1.00		
≥ 3	0.86	0.59–1.25	0.425

95% CI: 95% confidence interval

*Cox regression model adjusted for sex, age group, economic class, polypharmacy, potentially inappropriate medications (PMI) use, diabetes mellitus, arterial hypertension, and number of diseases.

**Wald's chi-square test.

**Fig 1:**
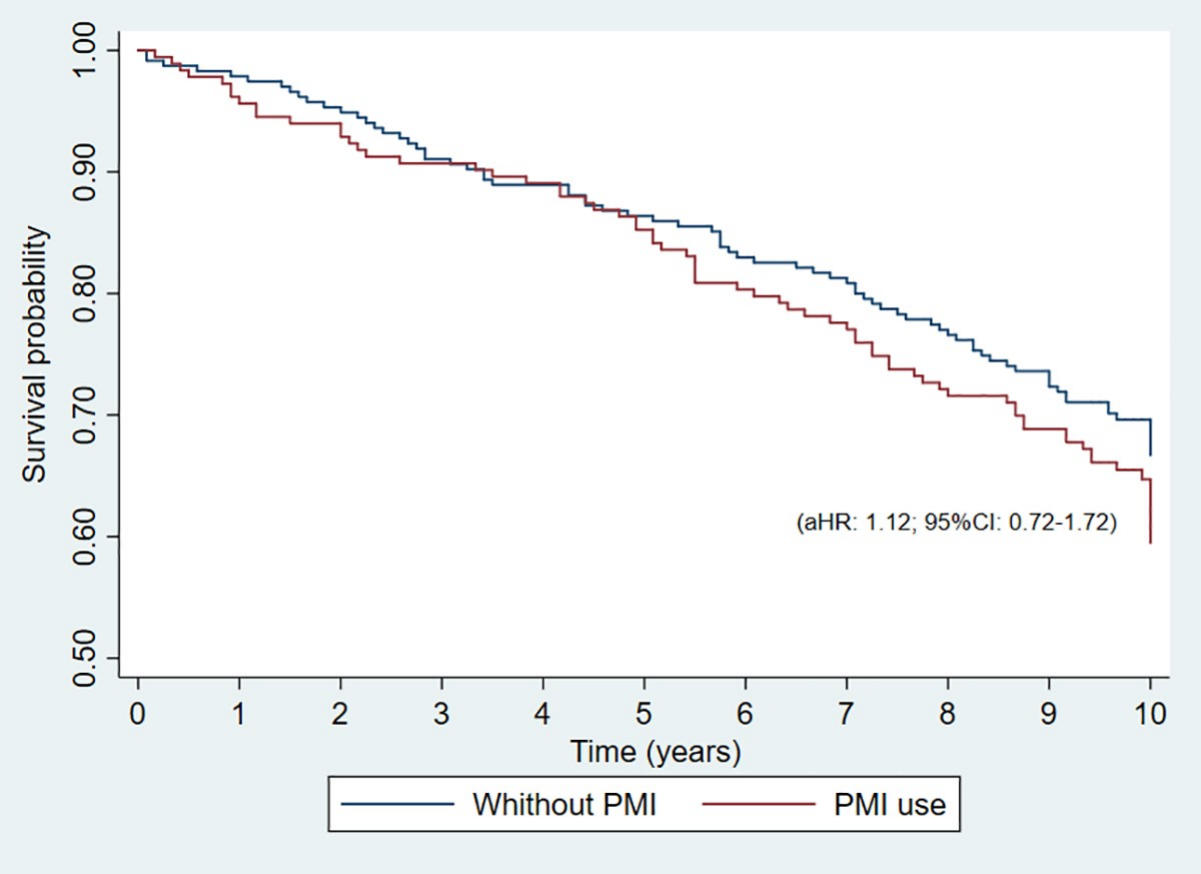
Probability of survival of the elderly according to statistically significant variables in the Cox proportional multiple regression analysis.

However, there was no statistical association between survival and the use of PIM in the adjusted analysis (aHR: 1.12; 95% CI: 0.72–1.72) (Table 6 & Fig 1).

## Discussion

The findings of this study showed the elderly undertake a high consumption of medications, including PIMs. The highest risk factors associated with the use of PIM were polypharmacy and diabetes mellitus. There was no statistically significant association between survival and the use of PIM. However, the use of polypharmacy significantly decreased survival.

The incidence of PIM use in this study was high. Previous studies conducted with elderly Brazilians have shown similar prevalence, ranging from 43.8% to 59.2% in the Southeast region [7, 17, 27, 30]. In the Northeast, Midwest and South of Brazil, the prevalence was lower, 21.6% [16], 26.0% [8], and 42.4% [2], respectively. International studies have shown prevalence rates of 43% in Canada [15], 42,7% in New Zealand [11], and 46.1% in Portugal [41]. In Asian countries, the prevalence was 12.7% in Oman [42], 34.0% in India [43], 39.4% in Indonesia [44], 53,1% in Kuwait [14], and 59.3% in Thailand [45]

These variations can be attributed to factors such as disease distribution in Brazil, the prescriber's specialty, and the version of the Beers criteria used (it is constantly updated with the insertion or removal of certain medications) [5, 27]. Most of the studies cited used versions prior to 2019. A study of elderly people in the public health system showed discrepancies between the Beers criteria of 2003 and 2012, with PIM proportions varying with the criteria used [27]. Furthermore, it should be noted that the Beers criteria was developed for the American profile, which limits its application in Brazil.

Regarding the types of drugs used, there was a high frequency in the consumption of central alpha-agonists, especially nifedipine and amiodarone, a result consistent with previous studies [7, 8, 46]. Nifedipine has the potential to cause hypotension and a risk of myocardial ischemia [5]. Conversely, amiodarone is associated with thyroid diseases, lung disorders, and the prolongation of the QT interval [5].

Glibenclamide also presented an elevated use in this study, as already observed in other studies [2, 28]. The class of sulfonylureas is one of the drugs that presents a higher risk of severe prolonged hypoglycemia in the elderly [5, 47–50]. Hypoglycemic agents are also high-risk medications, which can cause hypoglycemia and increase the incidence of acute myocardial infarction, stroke, and falls [51, 52].

There was a high frequency in the use of diclofenac sodium. Previous studies show that it is a medication mostly consumed by the elderly [2, 27], but it can increase the risk of gastrointestinal bleeding, leading to the development of peptic ulcers, in addition to increasing the risks for renal, heart failure, and hypertension [5, 47, 50].

Polypharmacy and diabetes mellitus were risk factors in the use of PIM. The relationship between polypharmacy and the high incidence of PIM observed is already well established in national and international literature [2, 16, 27, 53–55]. Polypharmacy is common among the elderly, owing to both multimorbidity and a frequent demand for health services. It is understood that chronic diseases appear during the fourth and fifth decades of life; often these drugs are not adjusted once patients are over 60 years of age [2]. The literature highlights the importance of qualifying clinical protocols and continuing education for the prescribing professionals in order to avoid excessive medications [16] as polypharmacy is associated with worsening physical and mental health conditions in the elderly population. Owing to its potential to cause harm to the patient, the World Health Organization underscores polypharmacy as one of the three priority categories of the Third Global Patient Safety Challenge [56].

A diagnosis of diabetes mellitus was a risk factor in the use of PIM. Some studies have shown that elderly people with diabetes mellitus consume multiple medications in greater proportions [57–61], which consequently may increase the risk of PIM use. It is noteworthy that some popular oral antidiabetics (such as glibenclamide and glimepiride) are considered inappropriate [5]. However, this relationship can be explained by the complexities of diabetes mellitus. Data from the Longitudinal Study of Health of Elderly Brazilians that was conducted in 70 municipalities of Brazil, showed that diabetes mellitus ranked seventh in diseases with a higher proportion among the elderly. Furthermore, it was the disease with the highest number of associated comorbidities, which in turn increases the number of drugs used for its control [57]. In addition, it causes varying complications. The symptoms or systemic manifestations of the complications can lead to the use of health services. Consequently, this leads to the use of other medications including PIMs.

Regarding mortality, the use of PIM did not increase the risk of death from any cause. However, polypharmacy was associated with mortality in the elderly. Most studies on medication use show that polypharmacy increases the risk of death in the elderly [18–21]. Nevertheless, the association between PIM and death is still controversial, owing to the classification criteria adopted and the length of time required for follow-up assessments. A recent study conducted in Japan comparing the risk of death of elderly people using PIM by two criteria, showed that the risk of death in five years of follow-up was 3.01 (95% CI 1.37–6.64) using the STOPP-J criteria, while using the Beers-Fick criteria it was 1.18 (95% CI 0.56–2.49) [62]. Likewise, other studies using the Beers criteria for PIM also found no association with death. This was demonstrated in Belgium with community-dwelling elderly and in the United Kingdom with elderly with frailty and elderly after hospital discharge in the United Kingdom [21, 63, 64]. In Brazil, a 15-year follow-up study showed that the risk of death among users of at least one PIM was 44% higher than those who did not use any PIM [18].

These results should be interpreted with caution, owing to the long follow-up period involved, other comparable studies (investigating the risk of death associated with the use of PIM) involved much shorter follow-up periods. In contrast, a study with a shorter follow-up period also showed an association between death and use of PIM. In Finland, a longitudinal study of 20,666 community-dwelling elderly found that the association of PIM and mortality was more significant in the first years of follow-up [65].

The main limitation of this study is the limited number of participants in the follow-up assessment. However, many cohort studies have already reported this limitation, especially in those conducted with individuals from the community who frequently move or refuse to continue the follow-up program. The Beers criteria used for the classification of PIM also presents limitations, since it was designed for North Americans. However, there are strengths such as the study design, follow-up time, and a large representative sample of the population.

The high incidence of PIM identified in this study reinforces that this is a persistent problem in health services and emphasizes the need for actions to reduce their use. De-prescription is one of the strategies proposed to reduce polypharmacy, through the identification and discontinuation of unnecessary, ineffective, unsafe, or potentially inappropriate medications [66]. In order to ensure de-prescription is effective in clinical practice, it is necessary to involve a multidisciplinary team to identify the potential harm of the medication to the patient. Assessment of each medication needs to include the objectives of the treatment, life expectancy, convenience, and preferences that may contribute to treatment adherence [67].

We recommend future studies that assess the barriers to PIM dissemination and implementation by health professionals, both in primary care as well as in other levels of care. Studies are also recommended to elaborate a national criteria specific to Brazil, which considers the local scenario, both in terms of drugs available in the country and the epidemiological context of diseases in the elderly population.

## Supporting information

S1 File Dataset. (DTA)

## References

[1] Borja-Oliveira CR, Assato CP. Psicofármacospotencialmenteinapropriados para idosos. Estudosinterdisciplinaressobre o envelhecimento [internet]. 2015; 20(3):687–70. https://seer.ufrgs.br/revenvelhecer/article/view/38548. Accessed 22 Jan 2019.

[2] Lutz BH, Miranda VIA, Bertoldi AD. Potentially inappropriate medications among older adults in Pelotas, Southern Brazil. Rev SaúdePública. 2017; 51:52; 10.1590/S1518-8787.2017051006556 28658367 PMC5493363

[3] Reeve E, Low LF, Hilmer SN. Attitudes of Older Adults and Caregivers in Australia toward deprescribing. J Am Geriatr Soc. 2019; 67(6):1204–10; 10.1111/jgs.15804 30756387

[4] Jafari F, Khatony A, Rahmani E. Prevalence of Self-Medication Among the Elderly in Kermanshah-Iran. Glob J Health Sci. 2015; 7(2); 10.5539/gjhs.v7n2p360 25716414 PMC4796481

[5] American Geriatrics Society Beers Criteria Update Expert Panel. American Geriatrics Society 2019 Updated AGS Beers Criteria for Potentially Inappropriate Medication Use in Older Adults. [59]. 2019; 67(4):674–94; 10.1111/jgs.15767 30693946

[6] Gallo C, Vilosio J, Saimovici J. Actualización de loscriterios STOPP-START: una herramienta para ladetección de medicaciónpotencialmenteinadecuadaenancianos. Evidencia, actualizaciónenlaprácticaambulatória [internet]. 2015; 18(4):124–29.http://www.evidencia.org/index.php/Evidencia/article/view/486. Accessed 15 Feb 2020.

[7] Cassoni TCJ, Corona LP, Romano-Lieber NS, Secoli SR, Duarte YAO, Lebrão ML. Use of potentially inappropriate medication by the elderly in São Paulo, Brazil: SABE Study. Cad SaúdePública. 2014; 30(8):1708–20; 10.1590/0102-311X00055613 25210910

[8] Santos TRA, Lima DM, Nakatani AYK, Pereira LV, Leal GS, Amaral RG. Medicine use by the elderly in Goiania, Midwestern Brazil. Rev SaúdePública. 2013; 47(1):94–103; 10.1590/s0034-89102013000100013 23703135

[9] Fernandez-Llimós F, Cabrita J, Morais J. Critérios de avaliação da prescrição de medicamentospotencialmenteinapropriados no doentegeriátrico: comparação de resultadospráticos. Revista Portuguesa de Farmacoterapia. 2015; 5(1):4–10; 10.25756/rpf.v5i1.56

[10] Masnoon N, Shakib S, Kalisch-Ellett L, Caughey GE. What is polypharmacy? A systematic review of definitions. BMC Geriatrics. 2017; 10.1186/s12877-017-0621-2 29017448 PMC5635569

[11] Nishtala PS, Bagge ML, Campbell AJ, Tordoff JM. Potentially inappropriate medicines in a cohort of community-dwelling older people in New Zealand. Geriatrics&GerontologyInternational. 2015; 14(1):89–93; 10.1111/ggi.12059 23530567

[12] Fadare JO, Agboola SM, Opeke OA, Alabi RA. Prescription pattern and prevalence of potentially inappropriate medications among elderly patients in a Nigerian rural tertiary hospital. Ther Clin Risk Manag. 2013; 9:115–20; 10.2147/TCRM.S40120 23516122 PMC3601648

[13] Patel T, Slonim K, Lee L. Use of potentially inappropriate medications among ambulatory home-dwelling elderly patients with dementia: A review literature. Canadian Pharmacists Journal. 2017; 150(3):169–83; 10.1177/1715163517701770 28507653 PMC5415067

[14] Awad A, Hanna O. Potentially inappropriate medication use among geriatric patients in primary care setting: a cross-sectional study using the Beers, STOPP, FORTA and MAI criteria. Plos One. 2019; 14(6):e0218174; 10.1371/journal.pone.0218174 31194800 PMC6563997

[15] Roux B, Sirois C, Simard M, Gagnon ME, Laroche ML. Potentially inappropriate medications in older adults: a population-based cohort study. Family Practice. 2019; 10.1093/fampra/cmz060 31602472

[16] Neves SJF, Marques APO, Leal MCC, Diniz AS, Medeiros TS, Arruda IKG. Epidemiology of medication use among the elderly in an urban area of Northeastern Brazil. Rev SaúdePública. 2013; 47(4):759–68; 10.1590/S0034-8910.2013047003768 24346667

[17] Oliveira MA, Francisco PMSB, Costa KS, Barros MBA. Automedicaçãoemidososresidentesem Campinas, São Paulo, Brasil: prevalência e fatoresassociados. Cadernos de SaúdePública. 2012; 28(2):335–45; 10.1590/S0102-311X2012000200012 22331159

[18] Nascimento MMG, Mambrini JVM, Lima-Costa MF, Firmo JOA, Peixoto SWV, Loyola Filho AI. Potentially inappropriate medications: predictor for mortality in a cohort of community-dwelling older adults. Eur J Clin Pharmacol. 2017; 73:615–621; 10.1007/s00228-017-2202-x 28108781

[19] Huang CH, Umegaki H, Watanabe Y, Kamitani H, Asai A, Kanda S, et al Potentially inappropriate medications according to STOPP-J criteria and risks of hospitalization and mortality in elderly patients receiving home-based medical services. Plos one. 2019; 8;14(2):e0211947; 10.1371/journal.pone.0211947 30735544 PMC6368320

[20] Paque K, Elseviers M, Stichele VR, Dilles T, Pardon K, Deliens L, et al Associations of potentially inappropriate medication use with four year survival of an inception cohort of nursing home residents. Arch gerontol geriatric. 2019; 80:82–87; 10.1016/j.archger.2018.10.011 30390429

[21] Porter B, Arthur A, Savva GM. How do potentially inappropriate medications and polypharmacy affect mortality in frail and non-frail cognitively impaired older adults? A cohort study. BMJ Open. 2019; 9:e026171; 10.1136/bmjopen-2018-026171 31092652 PMC6530304

[22] De Vincentis A, Gallo P, Finamore P, Pedone C, Costazo L, Pasina L, et al Potentially Inappropriate Medications, Drug–Drug Interactions, and Anticholinergic Burden in Elderly Hospitalized Patients: Does an Association Exist with Post-Discharge Health Outcomes? Drugs Aging. 2020; 10.1007/s40266-020-00767 32445121

[23] Huang Y, Zhang L, Huang X, Liu K, Yu Y, Xiao J, et al Potentially inappropriate medications in Chinese community dwelling older adults. Int J Clin Pharm. 2020; 42:598–603. 10.1007/s11096-020-00980-y 32026350

[24] Lu WH, Wen YW, Chen LK, Hsiao FY. Effect of polypharmacy, potentially inappropriate medications and anticholinergic burden on clinical outcomes: A retrospective cohort study.*Can Med J*. 2015; 187(4): e130–e137; 10.1503/cmaj.141219 25646290 PMC4347788

[25] Vegesna A, Alcusky MJ, Keith SW, Hegarty SE, Del Canale S, Lombardi M, et al Is there an association between potentially inappropriate prescribing in the elderly and hospitalization and mortality? A longitudinal, large cohort study.Value Health. 2015; 18: A86; https://core.ac.uk/reader/82408158

[26] Obreli-Neto PR, Cumam RKN. Medicamentospotencialmenteinapropriados para idosos e suapresença no SUS: avaliação das listaspadronizadas. Rev Bras GeriatrGerontol. 2011; 14(2):285–289; 10.1590/s1809-98232011000200009

[27] Baldoni AO, Ayres LR, Martinez EZ, Dewulf NLS, Santos V, Pereira LRL. Factors associated with potentially inappropriate medications use by the elderly according to Beers criteria 2003 and 2012. Int J Clin Pharm. 2014; 36(2):316–24; 10.1007/s11096-013-9880-y 24271923

[28] Lopes LM, Figueiredo TP, Costa SC, Reis AMM. Use of potentially inappropriate medications by the elderly at home. CiêncSaúde Colet. 2016; 21(11):3429–38; 10.1590/1413-812320152111.14302015 27828576

[29] Martins GA, Acurcio FA, Franceschini SCC, Priore SE, Ribeiro AQ. Use of potentially inappropriate medications in the elderly in Viçosa, Minas Gerais State, Brazil: a population-based survey.CadSaúde Pública.2015; 31(11):2401–12; 10.1590/0102-311X00128214 26840819

[30] Novaes PH. Comparação de critérios para avaliação de medicamentospotencialmenteinapropriados para idosos [dissertation]. Juiz de Fora: Universidade Federal de Juiz de Fora, Faculdade de Medicina; 2016. 9–120.

[31] Bueno D, Almeida TT, Rocha BS. Prevalência de prescrição de medicamentospotencialmenteinapropriados para idososemumaunidade de saúde da família de Porto Alegre/RS. Rev APS [internet]. 2016; 19(3):370–5. https://aps.ufjf.emnuvens.com.br/aps/article/view/2471.

[32] Silveira EA, Ferreira CCC, Pagotto V, Carvalho ASA, Velasquez-Melendez G. Total and central obesity in elderly associated with a marker of undernutrition in early life-sitting height-to-stature ratio: A nutritional paradox. Am J Hum Biol. 2017; 29(3); 10.1002/ajhb.22977 28161905

[33] Pagotto V, Santos KF, Malaquias SG, Bachion MM, Silveira EA. Calf circumference: clinical validation for evaluation of muscle mass in the elderly. Rev Bras Enferm. 2018; 71(2):322–328; 10.1590/0034-7167-2017-0121 29412289

[34] Folstein MF, Folstein SE, McHugh PR. Mini-mental state: a practical method for grading the cognitive state of patients for the clinician. J Psychiatr Res. 1975; 12(3):189–98; 10.1016/0022-3956(75)90026-6 1202204

[35] ABEP—ASSOCIAÇÃO BRASILEIRA DE EMPRESAS DE PESQUISA. CritérioBrasil: Critério de ClassificaçãoEconômicaBrasil–Base: PNADC 2017 2018 Disponívelem:http://www.abep.org/criterio-brasil.

[36] Pagotto V, Nakatani AYE, Silveira EA. Fatoresassociados à autoavaliação de saúderuimemidososusuários do sistemaúnico de saúde. Cad SaúdePública. 2011; 27(8):1593–602; 10.1590/S0102-311X2011000800014 21877007

[37] Malachias MVB, Souza WKSB, Plavnik FL, Rodrigues CIS, Brandão AA, Neves MFT et al 7ª Diretrizbrasileira de hipertensão arterial. ArqBrasCardiol. 2016; 107(3supl.3):1–83.

[38] SociedadeBrasileira de Diabetes. Diretrizes da SociedadeBrasileira de Diabetes 2017–2018. São Paulo: Clannad, 2017 https://www.diabetes.org.br/profissionais/images/2017/diretrizes/diretrizes-sbd-2017-2018.pdf. Accessed 22 Jan 2019.

[39] Wang YJ, Chiang SC, Lee PC, Chen YC, Chou LF, Chou YC, et al Is Excessive Polypharmacy a Transient or Persistent Phenomenon? A Nationwide Cohort Study in Taiwan. Frontiers in Pharmacology. 2018; 9:120; 10.3389/fphar.2018.00120 29515446 PMC5826280

[40] Lipschitz DA. Screening for nutritional status in the elderly. Prim Care. 1994; 21:55–67 8197257

[41] Simões PA, Santiago LM, Maurício K, Simões JA. Prevalence Of Potentially Inappropriate Medication In The Older Adult Population Within Primary Care In Portugal: A Nationwide Cross-Sectional Study. Patient Preference and Adherence. 2019; 13:1569–76; 10.2147/PPA.S219346 31571839 PMC6756837

[42] Al-Busaidi S, Al-Kharusi A, Al-Hinai M, Al-Zakwani I, Al-Ghafri F, Rizvi S, et al Potentially Inappropriate Prescribing among Elderly Patients at a Primary Care Clinic in Oman.J Cross Cult Gerontol. 2019; 10.1007/s10823-019-09393-5 31776821

[43] Bhatt AN, Paul SS, Krishnamoorthy S, Baby BT, Mathew A, Nair BR. Potentially inappropriate medications prescribed for older persons: A study from two teaching hospitals in Southern India. Journal of Family & Community Medicine. 2019; 26(3):187–92; 10.4103/jfcm.JFCM_81_19 31572049 PMC6755761

[44] Abdulah R, Insani WN, Destiani DP, Rohmaniasari N, Mohenathas ND, Barliana MI.Polypharmacy leads to increased prevalence of potentially inappropriate medication in the Indonesian geriatric population visiting primary care facilities. Ther Clin Risk Manag. 2018; 14(2):1591–97; 10.2147/TCRM.S170475 30233194 PMC6129028

[45] Vatcharavongvan P, Puttawanchai V. Potentially inappropriate medications among the elderly in primary care in Thailand from three different sets of criteria. Pharm Pract. 2019; 17(3):1494; 10.18549/PharmPract.2019.3.1494 31592037 PMC6763309

[46] Cuentro VS, Andrade MA, Gerlack LF, Bos AJG, Silva MVS, Oliveira AF. Prescriçõesmedicamentosas de pacientesatendidos no ambulatório de geriatria de um hospital universitário: estudo transversal descritivo. CienSaúde Colet. 2014; 19(8):3355–64. 10.1590/1413-81232014198.09962013 25119075

[47] Oliveira MG, Amorim WW, Borja-Oliveira CR, Coqueiro HL, Gusmão LC, Passo LC. Brazilian consensus of potentially inappropriate medication for elderly people. GeriatrGerontol Aging. 2016; 10(4):168–181; 10.5327/Z2447-211520161600054

[48] Faria AI, Obreli-Neto PR, Guidoni CM, Baldoni AO. Análise dos medicamentospotencialmenteinapropriados para idososcontidosnaRelação Municipal dos MedicamentosEssenciais (Remume) de Divinópolis-MG. JournalofAppliedPharmaceuticalSciences. 2015; 2(1):48–69.

[49] Secoli SR, Marquesini EA, Fabretti SC, Corona LP, Romano-Lieber NS. Self-medication practice trend among the Brazilian elderly between 2006 and 2010: SABE Study. RevBrasEpidemiol. 2012; 21(Suppl2):e180007; 10.1590/1980-549720180007.supl.2 30726352

[50] Garske CCD, Cassol D, Morch LM, Schneider APH. Medicamentospotencialmenteinapropriados para idososdispensados por umafarmáciabásica do sul do Brasil. RevistaInterdisciplinar de Promoção da Saúde. 2018; 1(2):96–104; 10.17058/rips.v1i2.12586

[51] Botosso RM, Miranda EF, Fonseca MAS. Reaçãoadversa medicamentosa emidosos. RevBrasCiêncEnvelhecHum. 2011; 8(2):285–97; 10.5335/rbceh.2012.1202

[52] Silva AS, Maciel GA, Wanderley LSL, Wanderley AG. Drug use indicators in primary health care: a systematic review. RevistaPanamericana de SaludPublica. 2017; 41:1–12; 10.26633/RPSP.2017.132

[53] Almeida NA, Reiners AAO, Azevedo RCS, Silva AMC, Cardoso JDC, Souza LC. Prevalence of and factors associated with polypharmacy among elderly persons resident in the community. Rev Bras GeriatrGerontol. 2017; 20(1):138–48; 10.1590/1981-22562017020.160086

[54] Abdulah R, Insani WN, Destiani DP, Rohmaniasari N, Mohenathas ND, Barliana MI. Polypharmacy leads to increased prevalence of potentially inappropriate medication in the Indonesian geriatric population visiting primary care facilities. TherClin Risk Manag. 2018; 14:1591–97; 10.2147/TCRM.S170475 30233194 PMC6129028

[55] Pereira KG, Peres MA, Iop D, Boing AC, Boing AF, Aziz M, et al. Polypharmacy among the elderly: a population-based study. RevBrasEpidemiol. 2017; 20(2):335–44; 10.1590/1980-5497201700020013 28832855

[56] WHO. The third WHO Global Patient Safety Challenge: Medication Without Harm. World Health Organization 2017 https://www.who.int/patientsafety/medication-safety/en/.Accessed 15 Feb 2020.

[57] Silva MRR, Diniz LM, Santos JBR, Reis EA, Mata AR, Araújo VE et al Drug utilization and factors associated with polypharmacy in individuals with diabetes mellitus in Minas Gerais, Brazil. Ciência&SaúdeColetiva. 2018; 23(8):2565–74; 10.1590/1413-81232018238.10222016 30137126

[58] Vitoi NC, Fogal AS, Nascimento CM, Franceschini SCC, Ribeiro AQ. Prevalence and associated factors of diabetes in the elderly population in Viçosa, Minas Gerais, Brazil. Rev Bras Epidemiol. 2015; 18(4):953–65; 10.1590/1980-5497201500040022 26982308

[59] Araujo MF, Freitas RW, Fragoso LV, Araújo TM, Damasceno MM, Zanetti ML. Oral antidiabetic drug therapy compliance among brazilian public health system users. Texto&ContextoEnferm. 2011; 20(1):135–43.

[60] Moraes SA, de Freitas IC, Gimeno SG, Mondini L. Prevalência de diabetes mellitus e identificação de fatoresassociadosemadultosresidentesemáreaurbana de ribeirãopreto, São Paulo, Brasil, 2006: ProjetoObediarp. Cad SaúdePública. 2010; 26(5):929–41. 10.1590/s0102-311x2010000500015 20563393

[61] Corallo F, Cola MC, Buono V, Lorenzo G, Bramanti P, Marino S. Observational study of quality of life of parkinson's patients and their caregivers. Psychogeriatrics. 2017; 17(2):97–102; 10.1111/psyg.12196 27338524

[62] Huang CH, Umegaki H, Watanabe Y, Kamitani H, Asai A, Kanda S et al Potentially inappropriate medications according to STOPP-J criteria and risks of hospitalization and mortality in elderly patients receiving home-based medical services. Plos One. 2019; 14(2):e0211947; 10.1371/journal.pone.0211947 30735544 PMC6368320

[63] Paque K, Vander Stichele R, Elseviers M, Pardon K, Dilles L, Deliens T et al Barriers and enablers to deprescribing in people with a life-limiting disease: A systematic review. Palliat Med. 2019; 33:37–48; 10.1177/0269216318801124 30229704

[64] Counter D, Millar JWT, McLay JS. Hospital readmissions, mortality and potentially inappropriate prescribing: A retrospective study of older adults discharged from hospital. British Journal of Clinical Pharmacology 2018; 84(8):1757–63; 10.1111/bcp.13607 29744901 PMC6046509

[65] Bo M, Quaranta V, Fonte G, Falcone Y, Carignano G, Cappa G. Prevalence, predictors and clinical impact of potentially inappropriate prescriptions in hospital-discharged older patients: A prospective study. Geriatr Gerontol Int. 2018; 18(4):561–8; 10.1111/ggi.13216 29265509

[66] Garfinkel D, Ilhan B, Bahat G. Routine deprescribing of chronic medications to combat polypharmacy. Ther Adv Drug Saf. 2015; 6 (6):212–33; 10.1177/2042098615613984 26668713 PMC4667766

[67] McGrath K, Hajjar ER, Kumar C, Hwang C, Salzman B. Deprescribing: A simple method for reducing polypharmacy. Journal of Family Practice [internet]. 2017; 66(7):436–45. https://www.ncbi.nlm.nih.gov/pubmed/287007583 28700758

